# Exploring the opportunities for food and drink purchasing and consumption by teenagers during their journeys between home and school: a feasibility study using a novel method

**DOI:** 10.1017/S1368980015000889

**Published:** 2015-04-15

**Authors:** Gill Cowburn, Anne Matthews, Aiden Doherty, Alex Hamilton, Paul Kelly, Julianne Williams, Charlie Foster, Michael Nelson

**Affiliations:** 1 British Heart Foundation Health Promotion Research Group, Nuffield Department of Population Health, University of Oxford, Old Road Campus, Headington, Oxford OX3 7LF, UK; 2 Physical Activity for Health Research Centre, Moray House School of Education, University of Edinburgh, Edinburgh, UK; 3 Public Health Nutrition Research (formerly Children’s Food Trust), London, UK

**Keywords:** School, Food and drink purchase and consumption, Adolescents, Wearable cameras, Methods

## Abstract

**Objective:**

To investigate the feasibility and acceptability of using wearable cameras as a method to capture the opportunities for food and drink purchasing/consumption that young people encounter on their regular journeys to and from school.

**Design:**

A qualitative study using multiple data-collection methods including wearable cameras, global positioning system units, individual interviews, food and drink purchase and consumption diaries completed by participants over four days, and an audit of food outlets located within an 800 m Euclidean buffer zone around each school.

**Setting:**

A community setting.

**Subjects:**

Twenty-two students (fourteen girls and eight boys) aged 13–15 years recruited from four secondary schools in two counties of England.

**Results:**

Wearable cameras offered a feasible and acceptable method for collecting food purchase and consumption data when used alongside traditional methods of data collection in a small number of teenagers. We found evidence of participants making deliberate choices about whether or not to purchase/consume food and drink on their journeys. These choices were influenced by priorities over money, friends, journey length, travel mode and ease of access to opportunities for purchase/consumption. Most food and drink items were purchased/consumed within an 800 m Euclidean buffer around school, with items commonly selected being high in energy, fat and sugar. Wearable camera images combined with interviews helped identify unreported items and misreporting errors.

**Conclusions:**

Wearable camera images prompt detailed discussion and generate contextually specific information which could offer new insights and understanding around eating behaviour patterns. The feasibility of scaling up the use of these methods requires further empirical work.

Concern over the nutritional adequacy of the habitual diets of children in England, alongside unprecedented levels of childhood obesity, has prompted interest in the impact of the wider food environment on the decisions children and young people take around food^(^ ^1^ ^–^ ^5^ ^)^. Although concepts of the food environment vary, definitions have generally been informed by social ecological models, which propose that health-related behaviours are influenced by a combination of factors that act at an individual, social, community and policy level^(^ ^6^ ^)^.

Children and young people mainly connect with the food environment at home and in school through visiting food outlets and via their exposure and receptiveness to food advertising and promotion. With increasing age, they have greater autonomy to interact with the food environment unsupervised by adults and begin to take more personal control of their food choices^(^ ^7^ ^–^ ^10^ ^)^. As evidence grows around the tracking of dietary intake between childhood and adulthood and its health implications^(^ ^11^ ^)^, it is important to understand the ways in which the interaction between the individual and the food environment take place in order to consider mechanisms for intervention which promote healthier food choices.

Recent food-based and nutrient-based standards for school food have made it easier for young people to make healthier food and drink choices at lunchtime and throughout the school day in England^(^ ^12^ ^–^ ^16^ ^)^. Beyond the school gate, however, little is known about what influences the food choices made by young people on their journeys between home and school. Evidence that the local food environment around schools has an impact on food purchase and consumption is equivocal^(^ ^17^ ^–^ ^20^ ^)^ although the presence of fast-food outlets near to schools has been viewed as providing ‘junk food temptation’ to young people, inconsistent with the healthy eating choices and messages received at school and potentially undermining the viability of school food services^(^ ^21^ ^)^.

In the present study, our main aim was to investigate the use of a novel method (a wearable camera, Vicon Revue) to assess its feasibility and acceptability as a research tool to capture and explore the opportunities for food and drink* purchasing and consumption that young people encounter on their regular journeys to and from school. The study was prompted by a policy context in which there has been a growing interest by local authorities in considering the restriction of access to particular types of food outlets near school premises^(^ ^22^ ^,^ ^23^ ^)^ and calls made for more reliable measures of the food environment^(^ ^24^ ^–^ ^26^ ^)^. An additional aim was to investigate whether Vicon Revue images could be used to aid recall of purchasing decisions and eating behaviour among young people, as wearable cameras had not previously been used with this age group. Because of the passive nature of image capture, wearable cameras may be a less-intrusive tool for data collection than ‘observational’ or traditional ‘photo-elicitation’ techniques, where the participants are required to interrupt their behaviour to take an image^(^ ^27^ ^)^. Wearable cameras have shown promise in studies aiming to improve assessment of dietary intake in adults^(^ ^28^ ^–^ ^30^ ^)^ and suggest that reactivity to wearing these devices is low^(^ ^31^ ^)^. Preliminary studies have shown that viewing wearable camera images provides powerful memory cues^(^ ^32^ ^)^ and interviews guided by photographs have provided unique insights around food purchasing behaviour in adults^(^ ^33^ ^)^. Alongside our interest in investigating whether wearable cameras were an acceptable research tool for use with adolescents, we were keen to test their practical application on a small scale to ascertain whether or not this novel method might hold some promise for future research^(^ ^34^ ^)^.

## Methods

### Sampling and recruitment

The selection of schools was driven by our assumption that school location might be an important factor influencing the food and drink purchasing behaviour of the young people in the study. With this in mind, we aimed to recruit from geographically diverse schools. We used Edubase (which classifies schools using the National Statistics Postcode Directory into geographical categories)^(^ ^35^ ^)^ to approach a purposive sample of six secondary schools from city, suburban and rural locations in Oxfordshire and Yorkshire. We invited these schools to take part in the study by letter to the head teacher, followed by a telephone call one week later. Head teachers selected a tutor group within the school to be involved.

Researchers visited the schools to explain the study to the nominated tutor group and to encourage individuals to consider taking part. Potential participants were blinded to our interest in their food and drink purchase and consumption. Individuals were told that the purpose of the research was to find out more about journeys to and from school from the student perspective: ‘where you go, who you go there with, what you do, and the reasons why you do the things you do’. Young people willing to take part were asked to gain signed parental consent. To find out whether using the wearable camera and other factors had influenced recruitment rates, we asked non-participants to complete a short feedback questionnaire, modified from one used by Caprani *et al*.^(^ ^36^ ^)^, about the recruitment process, which was administered by their tutor and returned by post.

Those with parental consent completed a screening tool so that a purposive sample could be selected, ensuring that young people using a range of typical forms of travel modes (walking, cycling, bus or car) to and from school were represented.

Our final sample included one city, two suburban and one village school, with one school declining because none of its students agreed to take part and the other declining for practical reasons.

### Data collection

**Fig. 1:**
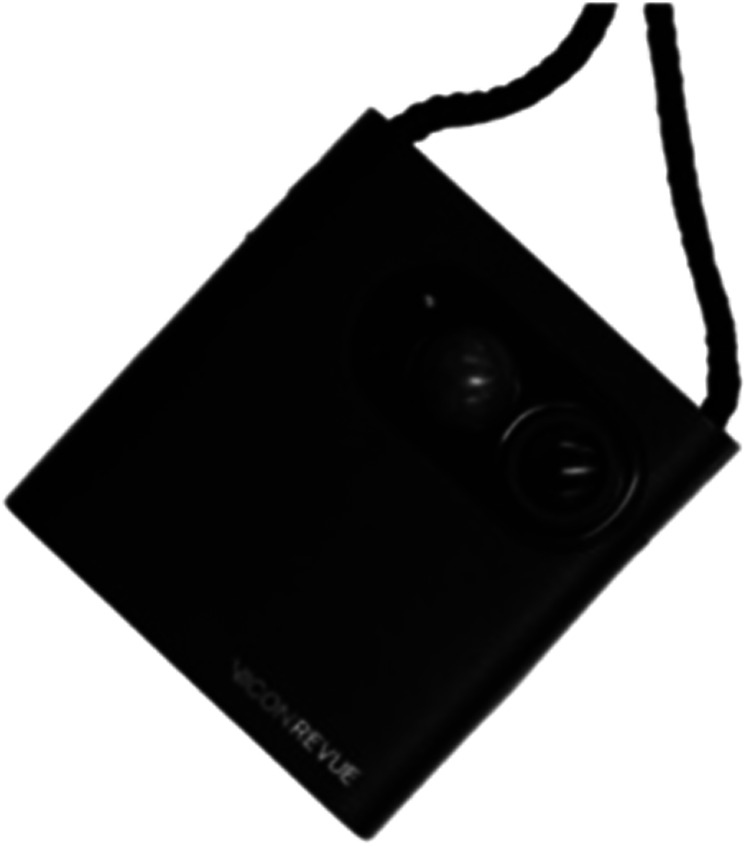
The Vicon Revue wearable camera

Data were collected between March and May 2011. We used the Vicon Revue wearable camera (version 1, shown in Fig. 1), which is worn on a lanyard around the neck. It automatically takes time-stamped, first-person point-of-view images every 10–15 s, without any action required from the wearer. In addition to time-stamped image capture, Vicon Revue contains a range of other sensors that trigger the taking of additional images when the wearer changes environment (for example, when the wearer goes from inside to outside a building or when movement is detected in front of the camera)^(^ ^37^ ^,^ ^38^ ^)^. There is also a privacy button on the camera, which users can press to stop image capture for 7 min. For the present study the images captured on the camera were encrypted so that they could only be viewed by the research team. We also used a Qstarz BT-Q1000XT Global Positioning System (GPS) device set to sample every 5 s^(^ ^39^ ^)^.

Each participant was shown how to use the technology and asked to wear Vicon Revue and to carry a GPS device over the next four consecutive school days. Although we were interested only in their journeys to and from school, participants were asked to wear the devices during the school day to accustom them and others to them, in an effort to reduce reactivity. Images collected during the school day were automatically deleted and were not available for analysis. Participants were told that they could remove the camera at any time if they wished or were asked to do so. They received text prompts before commencing their journey to school each morning and after they returned home at the end of the school day to remind them to charge the units and to wear them on their next journey to school.

At the beginning of the school day, each participant met with researchers who downloaded the data collected by the camera and GPS device from his/her journey to school that morning and his/her journey home from school the previous day. Participants completed a short paper-based questionnaire which recorded their travel mode and their food purchasing and consumption behaviour over the same period (see Appendix). Findings relating to the travel behaviour of the participants are published elsewhere^(^ ^40^ ^)^.

On the final day of data collection, each participant took part in an individual semi-structured interview. During the interview, participants viewed their complete set of Vicon Revue images and were offered the opportunity to delete any images if they wished to do so. The interviews used each set of camera images and questionnaire data as a starting point to explore, clarify and reflect on food purchasing and consumption behaviour of the participant during his/her journeys to and from school.

We also held a focus group in each school to explore food-related patterns in and around school and in relation to wider geographical and social factors. Participants in the focus group included the young people who had worn the data collection devices and other interested young people in the same tutor group who had received parental consent to take part but who were not selected to wear the camera and GPS unit. Interviews and focus groups were audio-recorded, with permission, for subsequent analysis.

To provide context for the study, we collected data about the school catering provision and observed food service in the main canteen, although we do not report this information here. Additionally, two researchers undertook an audit of all food outlets located within 400 m and 800 m Euclidean buffer zones^(^ ^41^ ^)^ around each school, using the Food Outlet Classification Tool described by Lake *et al*.^(^ ^42^ ^)^. An 800 m buffer size was chosen as it is commonly used in local authority planning and is a common measure of the region of influence in research about food access around schools^(^ ^17^ ^,^ ^43^ ^–^ ^46^ ^)^.

The study received ethics approval from the Social Sciences and Humanities Interdivisional Research Ethics Committee (IDREC) in accordance with the procedures laid down by the University of Oxford for ethical approval of all research involving human participants (IDREC reference number: SSD/CUREC1A/10-092).

### Data analysis

**Fig. 2:**
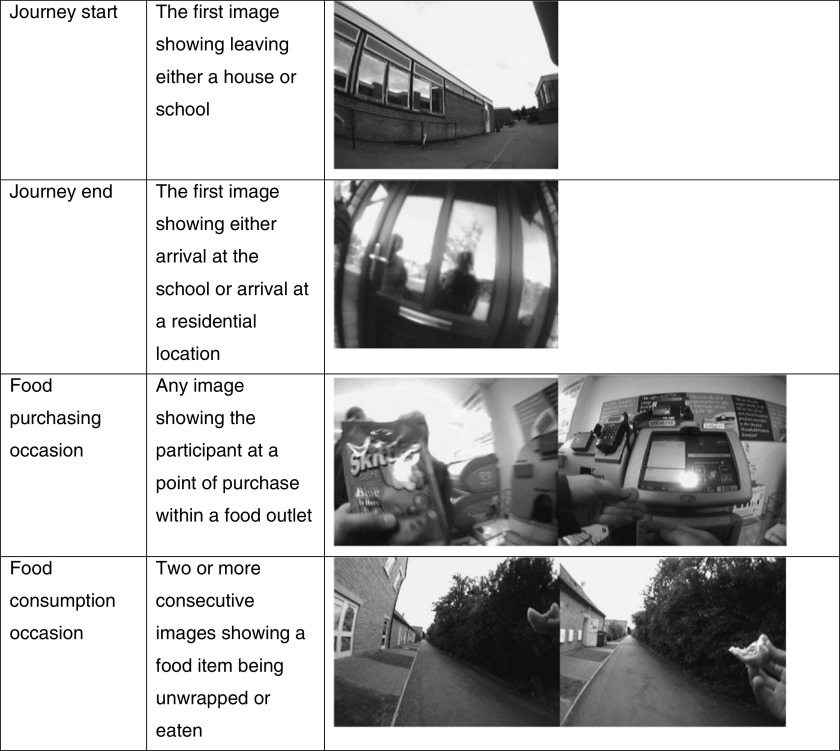
Definitions used during the analysis of the Vicon Revue images

Food purchase and consumption occasions were manually coded by two independent researchers from food diaries (G.C., A.M.) and using Vicon Revue images (G.C., A.H.). The camera images were viewed using standard software^(^ ^47^ ^)^. Figure 2 shows the definitions used during the analysis of the Vicon Revue images, with each occasion image being identified visually by the researcher^(^ ^40^ ^)^. The full protocol is available on request. An inter-rater reliability analysis using the kappa statistic was performed to determine consistency among researchers for identifying occasions of purchase or consumption during journeys. We uploaded GPS data to Google Earth^(^ ^48^ ^)^ to view participants’ GPS tracks. Using the time that food was purchased or consumed according to corresponding Vicon Revue data, we identified the participants’ GPS coordinates at the time of the event and calculated if it fell within an 800 m Euclidean buffer from the front entrance of the school.

## Results

**Fig. 3:**
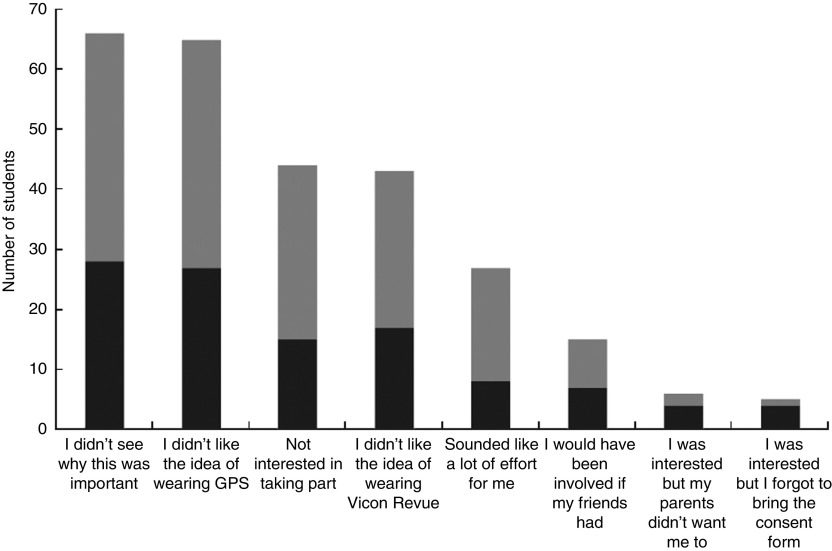
Reasons for non-participation among young people (*n* 70, respondents gave multiple reasons) who were invited but who chose not to participate in the study (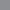, parent not involved in the decision; 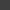, parent involved in the decision). GPS, Global Positioning System

A total of 125 young people were invited to participate in the study and thirty-three volunteered to take part (26 %), having gained parental consent. Of the ninety-two young people who we invited to take part who chose not to participate in the study, seventy completed non-participant questionnaires (76 % response rate). Figure 3 shows the reasons they gave for non-participation. Sixty-one per cent of non-participants indicated that issues relating to Vicon Revue played a part in their decision not to participate in the study (see Table 1).

**Table 1:** Reasons given for non-participation relating specifically to Vicon Revue (*n* 43, respondents gave multiple reasons)

Reason given for non-participation relating to Vicon Revue	Number	Typical comments
Invasion of privacy	16	‘It felt a little bit as if it was invading privacy’
		‘I could do things on the way to school I wouldn’t want people seeing’
		‘Because people would see my life and what I do’
Appearance	9	‘It looked a bit weird’
		‘Not attractive to wear’
		‘Too big and uncool’
Uncomfortable	5	‘It will be annoying having it round my neck all day’
Didn’t want to wear it	5	‘I didn’t want to walk round school with it on’
		‘I didn’t want to wear it all the time’
Fear of loss/breakage	5	‘I would probably lose it’
Parental concern	2	‘My parents didn’t like the idea of having a camera taking photos of me and my friends’
Other	3	‘I wouldn’t remember to wear it’
		‘I didn’t see the point…’
		‘It sounded odd’

Our final sample included twenty-two young people (fourteen girls and eight boys) aged 13–15 years old, with six participants recruited from each of the three suburban/village school settings and four participants from the one urban school setting. From the total of 176 possible journeys to and from school during the data collection period, 173 (98 %) food-related journeys were documented on diaries, with the missing data lost due to illness or absence from school. A total of 145 (82 %) sets of camera images were available for analysis, with the missing data lost due to equipment failure and user error. The inter-rater reliability between the food diaries and Vicon Revue images was good (*κ*=0·959, *P*<0·001 for purchase and *κ*=0·772, *P*<0·001 for consumption).

In total, thirty-eight items of food and drink were purchased or consumed by participants on their journeys to or from school. Most commonly these items were high in energy, fat and sugar: sixteen items (42 %) were categorised as chocolate/confectionery, eight items (21 %) were categorised as cakes and biscuits and seven items (18 %) were sweetened sugary beverages. Only four items of fruit were reported, with the remaining items categorised as crisps, a sandwich and tea.

All of these items were purchased or consumed by fifteen of the participants during twenty-eight of their journeys (16 % of possible opportunities). Most commonly, participants consumed food or drink items either purchased in school or brought from home (for example, left over from a packed lunch). Although food was purchased for consumption on the journey by some participants, we also found a few occasions of food being purchased for consumption at a later date, for example at home or at an after-school club. On eleven of the fourteen occasions when food was purchased from a food outlet, the participant was walking to or from school. Eight of the participants purchased or consumed food on their journey to or from school only once during the week of data collection, whereas the remaining seven were regular purchasers or consumers of food on their journeys (range 2–5 times). Seven young people did not report (and were not observed via Vicon Revue) purchasing or consuming any food or drink during any of their journeys to and from school. The reasons for not purchasing food varied. Some young people said that their journey did not pass conveniently close to a shop or that their transport mode meant they had no access to a food outlet. For others, purchasing power was an important issue – some reported a lack of spending money, others were owed money by their friends or were saving for more important items (like a new bicycle). As most journeys to and from school were quite short, some preferred to wait until they got home or to a friend’s house to eat food available there.

Table 2 shows the number of food outlets located within an 800 m Euclidean buffer zone around the participating schools. Only three of the food outlets were located within a smaller (400 m Euclidean) buffer zone of two of the schools: a convenience store/off-licence and a sandwich shop were located near to the city school; and the village school had a vending machine located at the on-site leisure centre, although a school policy prohibited access to the vending machine during school hours.

**Table 2:** Food outlets located within an 800 m Euclidean buffer zone around the participating schools

	Food outlets within 800 m Euclidean buffer zone		No. of participants purchasing or consuming food	No. of occasions of food purchase or
School location	Total	Type	on their journeys (*n* 15)	consumption (*n* 28)
City	14	Takeaway (*n* 4)	3	4
		Pub/bars (*n* 3)		
		Convenience – traditional (*n* 2)		
		Convenience – off-licence (*n* 3)		
		Sandwich shop (*n* 2)		
Suburban school 1	17	Specialist traditional – confectioners, greengrocer, baker, butcher (*n* 5)	3	4
		Restaurants (*n* 4)		
		Café/coffee shop (*n* 3)		
		Convenience – petrol station (*n* 2)		
		Fast food (*n* 1)		
		Supermarket – small multiple (*n* 1)		
		Vending machine (*n* 1)		
Suburban school 2	3	Takeaway (*n* 2)	5	15
		Supermarket – small multiple (*n* 1)		
Village	1	Vending machine	4	5

The location of twenty-seven of the thirty-eight food or drink items purchased or consumed during the school journey could be verified either by GPS or camera. Of these, fifteen (55 %) were purchased of consumed within the 800 m Euclidean buffer around the participating schools.

Most (64 %) of the purchase or consumption occasions were reported both on food diaries and confirmed by Vicon Revue images. In addition, Vicon Revue images located eight food purchase/consumption occasions that were not reported on the food diaries. The images proved useful in identifying both unreported items and in correcting misreporting errors, identified during the image review:

‘Now, I’ve got a chocolate bar – how did I not remember that?’ (Female, suburban school 1)

‘I think I went back in though… I think I went to get another drink, it was for the next day… I don’t actually think I wrote it down though, I put food but I didn’t put drink.’ (Male, suburban school 2)

We found that coding the camera images alongside the interview data was more helpful than viewing the images in isolation. This helped to avoid miscoding errors; in one example, what appeared on an image to be a purchase of a soft drink was corrected by the participant during the interview:

‘This is the shop, here. The ginger beer is on that corner, you can’t see it. And then I think it was me just looking then and then I came back and said do you want to get some and then he wanted to buy it so here we are with it, here’s the lady [shopkeeper]. I think he [friend] had the money and I was counting it, so I’ve taken the money but we didn’t have enough, well technically we weren’t 1p short because it was a cent, so we left.’ (Female 1, village school)

**Figure:**
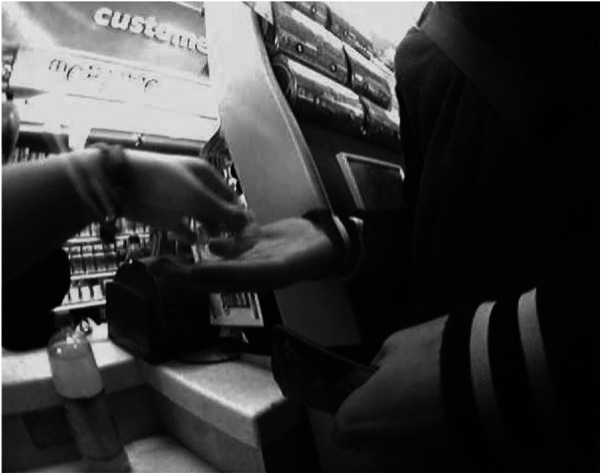

However, Vicon Revue also missed fourteen occasions of items being purchasing/consumed that participants did report via the food diary. This was in part due to equipment failure but also because the camera units used in the present study were early versions which capture low-resolution images too infrequently.

Although non-participants suggested that Vicon Revue was part of their rationale for non-involvement in the study, among participants there was little concern:

‘Some people don’t like their pictures being taken though, so when you’ve first got it on people say I don’t want to stand in front of you, my picture might get taken… people are worried that they might look bad… and are worried about their privacy – I don’t know why, they are only walking to school… I wasn’t worried because I know only a couple of people would see it and then they’d just get deleted anyway.’ (Focus group discussion, suburban school 2)

This lack of concern may have been to do with the participants’ particular personal qualities. During recruitment, teachers confirmed the general reliability of each participant and we found them to be confident individuals who may have been able to easily dismiss the reactions of others.

We did lose some data from user error but, generally, most of the participants successfully wore and managed the equipment:

‘Towards the end of the week it sort of became like a routine, take it out of my jumper when we were walking home, charge it when I got home, before I went to bed to swap it round [to charge the GPS unit] and put it on in the morning.’ (Female 2, village school)

Participants did comment about wearing the camera units but suggested that familiarity reduces reactivity:

‘To begin with you felt like you were being watched but then you just got used to it.’ (Female 3, village school)

‘It’s been alright, lots of people been asking questions and that but it’s been fine… after Wednesday it was alright, not so many people would be asking… I forgot it was there a few times.’ (Male, suburban school 1)

‘People tried getting in front of it, jumping around to get it to take pictures, like, they got used to it.’ (Female, city school)

## Discussion

To our knowledge, the present study is the first to explore the feasibility of using wearable cameras as a mechanism to understand food purchasing and consumption behaviour among teenage participants. We deliberately used a small, purposive sample of volunteers who were keen and willing to participate and, as such, we are unsure how likely they are to be ‘typical’ teenagers. Our findings should be viewed as a cautious first step, which hint at some promising opportunities to study the behaviour of this age group of participants in the future.

We faced some resource and practical issues which limit the study. We only had a small number of Vicon Revue units available for testing which constrained the number of participants we could recruit per school. We were using early models of the Vicon Revue units that capture an image every 10–15 s^(^ ^37^ ^,^ ^38^ ^)^, which is probably too infrequent to provide a reliably comprehensive record of behaviour. However, the newest version of the cameras (Vicon Autographer) captures higher-resolution pictures (5 megapixels *v*. 0·3 megapixels) and is likely to soon have the ability to adjust image capture rates down to 1 s intervals.

We did find some resistance to involvement in the study due to concerns about the use of the cameras but these concerns were less common among non-participants than the idea of using a GPS device. Mostly, these camera-specific issues related to reasonable concerns about privacy but several non-participants were also put off by the appearance of the wearable cameras, which were considered to be too big, bulky and unattractive. Newer versions of the cameras have been redesigned to look slimmer and sleeker, which may address some of these issues for future studies. Those teenagers who chose to participate proved to be reliable and diligent custodians of the (expensive) equipment and did not seem concerned about wearing the camera.

Researchers using wearable cameras have reported ethical concerns within the research community around the use of these devices^(^ ^49^ ^,^ ^50^ ^)^ but we found no difficulty in gaining ethical committee approval for our study, once safeguarding procedures had been put into place. Some non-participants cited their own (or parental) concerns over invasion of privacy, although participants demonstrated less concern about this. Future work is needed to examine how best to make use of wearable camera images in a respectful and ethical manner.

Although we had a higher rate of data loss using Vicon Revue compared with the food diary data, this was mostly due to equipment failure (again because we used early models of the units) rather than user error and we would suggest that wearable cameras seem to be a practical research tool for use with this age group. Unlike O’Loughlin *et al.* ^(^ ^29^ ^)^ who found some camera images to be of low quality in poorly lit conditions, we found image quality was generally good enough to establish occasions of purchase/consumption, presumably because our participants were travelling to and from school during spring-time, when light levels were satisfactory.

Arab *et al.* found that automated image capture using a camera-equipped mobile phone together with user-initiated image-assisted recall was a promising method for dietary assessment^(^ ^51^ ^)^. Gemming *et al.* found that a wearable camera can be used to reveal unreported foods and misreporting errors in their study to test whether wearable cameras could reduce under-reporting during self-reported dietary assessment^(^ ^28^ ^)^. Similarly, O’Loughlin *et al.* suggested that more valid and reliable estimates of total energy intake can be provided when wearable cameras are used alongside a conventional food diary^(^ ^29^ ^)^. Our findings suggest that using food records, wearable camera images and interview data in combination might assist in improving the validity and reliability of reporting of dietary behaviour, especially among girls in this age group, in whom under-reporting is widespread and large^(^ ^52^ ^)^. Wearable camera images viewed alone can present a coding challenge, particularly when searching for relatively infrequent short-duration behaviour episodes like eating snacks. The images did seem to facilitate vivid recall of particular incidents for our participants and using interview data in conjunction with images and food record diaries seems a fruitful way to help researchers and participants reach agreement about the details of the incident under discussion. This might prove valuable as a means of reducing unreported/misreported items and miscoding errors which are common features of dietary assessment^(^ ^28^ ^,^ ^29^ ^)^.

Our participants reported diminished concern about wearing the Vicon Revue units as the study progressed, suggesting that users become familiar and more comfortable with increasing time and exposure to wearable camera use. We found no differences in completion of the questionnaire across the study days, suggesting little detectable impact on the behaviour of the students. Other studies have reported varying degrees of awareness of wearable cameras^(^ ^28^ ^,^ ^51^ ^)^ and little is known about the impact of this awareness and habituation on usual behaviour.

Our own limited resources for this exploratory study meant that we did not systematically record or analyse the time spent on the analysis of the diaries and camera images. Using manual systems, it is both time-consuming and labour-intensive, and thus potentially expensive. It would be worth undertaking this analysis in a larger sample to help clarify the costs *v*. benefit of this type of manual analysis. Sun *et al.* have used a wearable electronic system incorporating elements of computer-led image recognition and analysis to assess dietary intake^(^ ^53^ ^)^. As computer-assisted analysis becomes more sophisticated, it may be possible to reduce researcher burden in future studies^(^ ^54^ ^)^.

Among our sample we found evidence of non-purchase/consumers, occasional and regular purchase/consumers. Participants were making deliberate choices about whether or not to purchase and consume food and drink on their journeys. These choices were influenced by a range of priorities over resources (such as money), social engagement (interactions with friends), length of journey, travel mode and ease of access to opportunities for purchase/consumption. However, the distance of a food outlet from school did not seem a strong influencing factor for our participants. Several participants did not engage with food-related behaviour on their journeys to and from school, despite having the opportunity. One explanation could be that their behaviour is framed by their travel routes and transport modes through the food environment. The freer participants were to go where they chose en route between home and school, the more likely they were to engage with the food environment around them. The food environment was also framed by school location: rural schools had fewer food opportunities than those in urban settings. Interpersonal factors relating to family structure and peer group socialising, and individual factors such as knowledge and attitudes towards health, money and food, all emerged as influential. Whether or not these patterns of behaviours would be found in a larger, more representative sample of teenagers requires further study. Because of the exploratory nature of the study, we did not set out to examine the contribution made by food purchased/consumed on the journey to and from school to the total dietary intake of participants.

Our small, proof-of-concept study has demonstrated the feasibility of using a wearable camera alongside more traditional methods of data collection to explore and elucidate the food purchasing and consumption behaviour of a small number of teenagers. Vicon Revue or similar devices seem to be a helpful tool, worth considering when the direct observation of behaviour may be inappropriate, impractical or intrusive. In particular, wearable camera images prompt detailed discussion and generate contextually specific information which could offer new insights and understanding around eating behaviour patterns.

Uniquely, the present study suggests a method to place exposure to food retail and actual purchasing and consumption behaviour of individuals in context. This offers the potential for greater precision to estimate real interactions with the food environment and move beyond the limitation seen in other studies where exposure to the food retail environment with food behaviour is assumed based on proximity of outlets within a buffer zone around schools.

## Footnotes

* Throughout the text we use ‘food’ to include food and drink.

## Appendix

***Questionnaire on travel mode and food purchasing and consumption behaviour***

**Table:**

School…………………………………………………..Student name…………………….……………………………. Date…………………………			
	Yesterday lunchtime	Yesterday’s journey home from school	This morning’s journey to school
**Tues**	Did you go out of school at lunch?	Did you go straight home from school?	Did you go straight to school from home?
	No □	Yes □	Yes □
	Yes □	No □	No □
	If Yes, where did you go and who with?	If No, where did you go and who with?	If No, where did you go and who with?
	Did you buy anything while out of school?	Did you buy anything on your way home from school?	Did you buy anything on your way from home to school?
	No □	No □	No □
	Yes □	Yes □	Yes □
	What did you buy?	What did you buy?	What did you buy?
	Did you eat anything while out of school?	Did you eat anything on your way home from school?	Did you eat anything on your way from home to school?
	No □	No □	No □
	Yes □	Yes □	Yes □
	What did you eat?	What did you eat?	What did you eat?
**Wed**	Did you go out of school at lunch?	Did you go straight home from school?	Did you go straight to school from home?
	No □	Yes □	Yes □
	Yes □	No □	No □
	If Yes, where did you go and who with?	If No, where did you go and who with?	If No, where did you go and who with?
	Did you buy anything while out of school?	Did you buy anything on your way home from school?	Did you buy anything on your way from home to school?
	No □	No □	No □
	Yes □	Yes □	Yes □
	What did you buy?	What did you buy?	What did you buy?
	Did you eat anything while out of school?	Did you eat anything on your way home from school?	Did you eat anything on your way from home to school?
	No □	No □	No □
	Yes □	Yes □	Yes □
	What did you eat?	What did you eat?	What did you eat?
**Thurs**	Did you go out of school at lunch?	Did you go straight home from school?	Did you go straight to school from home?
	No □	Yes □	Yes □
	Yes □	No □	No □
	If Yes, where did you go and who with?	If No, where did you go and who with?	If No, where did you go and who with?
	Did you buy anything while out of school?	Did you buy anything on your way home from school?	Did you buy anything on your way from home to school?
	No □	No □	No □
	Yes □	Yes □	Yes □
	What did you buy?	What did you buy?	What did you buy?
	Did you eat anything while out of school?	Did you eat anything on your way home from school?	Did you eat anything on your way from home to school?
	No □	No □	No □
	Yes □	Yes □	Yes □
	What did you eat?	What did you eat?	What did you eat?
**Fri**	Did you go out of school at lunch?	Did you go straight home from school?	Did you go straight to school from home?
	No □	Yes □	Yes □
	Yes □	No □	No □
	If Yes, where did you go and who with?	If No, where did you go and who with?	If No, where did you go and who with?
	Did you buy anything while out of school?	Did you buy anything on your way home from school?	Did you buy anything on your way from home to school?
	No □	No □	No □
	Yes □	Yes □	Yes □
	What did you buy?	What did you buy?	What did you buy?
	Did you eat anything while out of school?	Did you eat anything on your way home from school?	Did you eat anything on your way from home to school?
	No □	No □	No □
	Yes □	Yes □	Yes □
	What did you eat?	What did you eat?	What did you eat?
